# The Replication Stress Response on a Narrow Path Between Genomic Instability and Inflammation

**DOI:** 10.3389/fcell.2021.702584

**Published:** 2021-06-25

**Authors:** Hervé Técher, Philippe Pasero

**Affiliations:** Institut de Génétique Humaine, CNRS, Université de Montpellier, Equipe Labellisée Ligue Contre le Cancer, Montpellier, France

**Keywords:** DNA replication dynamics, fork progression, fork processing, fork reversal, cGAS-STING, inflammation

## Abstract

The genome of eukaryotic cells is particularly at risk during the S phase of the cell cycle, when megabases of chromosomal DNA are unwound to generate two identical copies of the genome. This daunting task is executed by thousands of micro-machines called replisomes, acting at fragile structures called replication forks. The correct execution of this replication program depends on the coordinated action of hundreds of different enzymes, from the licensing of replication origins to the termination of DNA replication. This review focuses on the mechanisms that ensure the completion of DNA replication under challenging conditions of endogenous or exogenous origin. It also covers new findings connecting the processing of stalled forks to the release of small DNA fragments into the cytoplasm, activating the cGAS-STING pathway. DNA damage and fork repair comes therefore at a price, which is the activation of an inflammatory response that has both positive and negative impacts on the fate of stressed cells. These new findings have broad implications for the etiology of interferonopathies and for cancer treatment.

## Introduction

**FIGURE 1:**
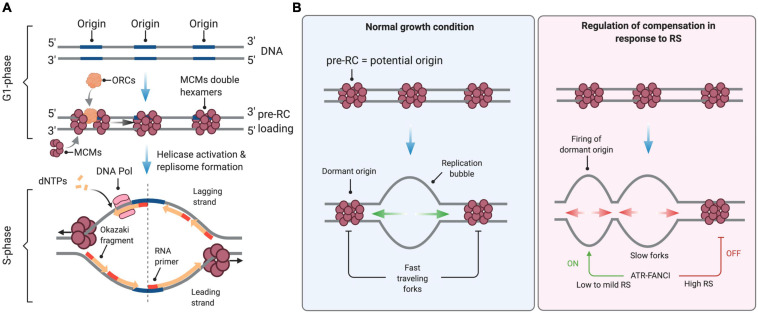
Control of DNA replication origin firing. **(A)** Origins of replication are licensed during the G1 phase of the cell cycle through the sequential loading of the ORC and MCM complexes to form the pre-replication (pre-RC) complex. At the S-phase onset, S-CDK and DDK activate the replicative helicase by recruiting CDC45 and the GINS complex, enabling DNA unwinding and the formation of active replisomes. dNTPs are the essential building blocks used by the replicative DNA polymerases to elongate nascent DNA. **(B)** In normal growth conditions, firing is limited to a few licensed origins. Forks travel at full speed, inactivating neighboring unfired origins. Under conditions of low to mild RS, cells compensate for slow elongation by activating backup origins known as dormant origins. Under acute RS conditions, the ATR-FANCI pathway restrains the activation of new origins. See the text for further details. This figure has been created with BioRender.com.

Eukaryotic DNA replication refers to a complex set of biological processes that duplicate chromosomal DNA during the S phase of the cell cycle. Briefly, DNA replication is pre-set in G_1_, when origins of replication are “licensed” through the assembly of the pre-replication complex (pre-RC) on chromatin (Mechali, 2010). During this process, the six-subunit origin recognition complex (ORC) provides a platform to load the minichromosome maintenance (MCM) complex in a Cdc6- and Cdt1-dependent manner (Figure 1A). Upon entry into S phase, cyclin-dependent kinases (CDKs), and Dbf4-Cdc7 (DDK) activate a subset of these potential replication origins (pre-RCs). A critical step is the formation and activation of the replicative helicase, namely the CMG (CDC45-MCMs-GINS) complex, which opens the DNA duplex, leading to the formation of a replication bubble onto which the replication machinery—composed of two replisomes—assembles and initiates DNA synthesis. A replication bubble is composed of two replication forks traveling in opposite directions. In metazoans, the nature of replication origins is still poorly understood. It appears that diverse cues at the level of the DNA sequence and chromatin conformation contribute to the establishment of the pool of potential origins and the efficiency of origin firing (Mechali, 2010; Hyrien, 2015; Urban et al., 2015; Valton and Prioleau, 2016).

During the S phase, hundreds to thousands of origins fire sequentially at defined times to ensure the completion of DNA replication before chromosome segregation. Origin firing follows a specific spatial and temporal program—not yet understood in the very details—that is cell-type specific and is determined epigenetically by the chromosome environment (Rivera-Mulia and Gilbert, 2016). In mammals, the timing and efficiency of origin activation correlates also with ORC density, as indicated by chromatin immunoprecipitation experiments (Miotto et al., 2016). Only a fraction of all licensed origins is used during a normal S phase. The pool of “licensed” but inactive origins, also known as dormant origins, serves as backup to complete DNA synthesis in case of fork slowing or stalling (Blow et al., 2011; Técher et al., 2017).

DNA replication is often challenged by events of exogenous or endogenous origin that impede the rate and fidelity of DNA synthesis, and as a consequence affect the integrity of chromosomes. These events are collectively referred to as replication stress (RS). They include DNA lesions caused by ultraviolet (UV) light or oxidative DNA damage, shortage of deoxyribonucleotides (dNTPs) or exposure to a broad panel of genotoxic agents used in chemotherapy to target DNA replication. In all eukaryotes, RS is detected and signaled through a conserved pathway involving the Mec1 and Rad53 kinases in budding yeast (Pardo et al., 2017) and the ATR and CHK1 kinases in mammals (Zeman and Cimprich, 2014). The ATR-CHK1 pathway senses and signals the presence of single-stranded (ss) DNA at impaired replication forks and on damaged DNA.

The mechanisms that regulate DNA replication during the cell cycle and coordinate the cellular responses to RS have been extensively discussed elsewhere (Mechali, 2010; Renard-Guillet et al., 2014; Zeman and Cimprich, 2014; Bellelli and Boulton, 2021). This review addresses the events that perturb the progression, the structure and the stability of replication forks, with a focus on the links between the stability of nascent DNA at stalled replication forks and the cellular responses to self DNA by the cGAS-STING pathway. Although we discuss conceptual advances obtained from model systems such as yeast and *Xenopus* egg extracts, this review focuses on mammalian models because of recent advances on the links between RS and innate immunity. This connection has major implications for cancer therapy by opening new avenues for the development of innovative strategies exploiting RS-induced inflammation.

## DNA Replication Under Normal and Pathological Conditions

### Normal Fork Progression

Eukaryotic replication forks progress at a median speed of 1–2 kb per min in unperturbed growth conditions (Tuduri et al., 2010; Guilbaud et al., 2011; Técher et al., 2013). Determination of fork speed was made possible with the development of DNA fiber autoradiography (Huberman and Riggs, 1966) and related immunofluorescence-based assays called DNA fiber spreading (Jackson and Pombo, 1998), DNA combing (Michalet et al., 1997) and SMARD (Single Molecule Analysis of Replicated DNA) (Norio and Schildkraut, 2001). In these assays, ongoing DNA synthesis is labeled with halogenated thymidine analogs and the length of labeled tracks is measured to determine the speed of individual replication forks.

Fork speed shows a broad distribution in a population of cells, reflecting either cell-to-cell differences or locus-specific variations. DNA synthesis requires a constant supply of histones (Groth et al., 2007; Mejlvang et al., 2014) and dNTPs (Anglana et al., 2003; Poli et al., 2012) that can fluctuate during S phase and thus could affect the overall speed of replication forks. In human HCT116 cells, forks are slower in early S phase than in late S phase (Malinsky et al., 2001; Bianco et al., 2019), presumably because dNTP levels are lower upon entry into S phase (Malinsky et al., 2001). This is reminiscent of *S. cerevisiae* cells, which enter S phase with suboptimal dNTP pools and activate the Mec1^*ATR*^ pathway to induce dNTP synthesis and complete bulk DNA synthesis (Forey et al., 2020). However, differences between early and late DNA synthesis were not observed in other cell types (Guilbaud et al., 2011; Eykelenboom et al., 2013). Further work is therefore needed to elucidate the complex interplay between replication timing, dNTP levels and fork speed.

In addition to global changes in replication rate, specific DNA sequences or chromosomal structures may locally impede fork progression. For instance, a variety of programmed replication pause sites have been identified in the genome of unicellular eukaryotes by two-dimensional gel electrophoresis (Deshpande and Newlon, 1996; Mirkin and Mirkin, 2007; Lambert and Carr, 2013). In large genomes, DNA fiber analysis is a method of choice to monitor site-specific events when combined with fluorescence *in situ* hybridization (FISH) to identify loci of interest along individual DNA fibers. This strategy was used to compare fork speed at specific loci relative to bulk DNA synthesis (Norio and Schildkraut, 2001; Pasero et al., 2002; Lebofsky et al., 2006) and led to contrasting results. Forks do not slow down at difficult-to-replicate loci such as the common fragile site (CFS) FRA3B (Letessier et al., 2011) and at the immunoglobulin heavy chain (IgH) locus (Guilbaud et al., 2011). However, they are slower at the AT-rich FRA16C CFS (Ozeri-Galai et al., 2011) and stall at CGG/CCG trinucleotide repeats located at the FMR1/fragile X locus (Gerhardt et al., 2014). Reduced fork velocity is also observed at centromeric and pericentromeric regions containing G4 (G-quadruplex)-forming sequences (Mendez-Bermudez et al., 2018), which are susceptible to breakage under replication stress conditions (Crosetto et al., 2013). Together, these studies indicate that although non-B DNA structures could locally slow down fork progression at specific loci, the mechanisms that govern the overall distribution of fork velocity in a population of cells remain poorly understood. At the level of the FRA3B CFS, completion of replication is challenged by the late timing of replication of this region and the lack of replication origins (Letessier et al., 2011). Difficult-to-replicate loci, such as some CFS, are thus not only defined by DNA sequence-driven impediments to fork passage, other important cues include replication timing, availability of replication origins, proficiency for restart and repair mechanisms, all of which being impacted by specific chromatin context. Interestingly, it has been recently reported that identical DNA molecules replicated *in vitro* by reconstituted replisomes also show a wide distribution of fork speed (Graham et al., 2017; Kurat et al., 2017; Yeeles et al., 2017), suggesting that replication forks stochastically pause and restart in a locus-independent manner.

### Regulation of Fork Progression Under Replication Stress

Conditions that slow down or block replication fork progression are collectively referred to as RS (Zeman and Cimprich, 2014; Bellelli and Boulton, 2021). RS is often detected indirectly by measuring the activity of the ATR-CHK1 pathway (Toledo et al., 2017). It can also be more directly assessed with DNA fiber assays by comparing the length of replicated tracks before and after exposing cells to exogenous inducers of RS (Técher et al., 2013; Toledo et al., 2017). The impact of RS on fork velocity has been extensively documented by many groups. For example, fork progression is dramatically impacted by DNA alkylating agents (Merrick et al., 2004) and by hydroxyurea (HU), an inhibitor of ribonucleotide reductase (RNR) inducing dNTP starvation (Técher et al., 2016, 2017). Aphidicolin (APH), an inhibitor of replicative DNA polymerases, is also commonly used to slow down DNA synthesis (Koundrioukoff et al., 2013). Although, slowdown of replication forks is probably the most direct manifestation of RS, which can be directly assessed with DNA fiber analysis, there are other causes of RS, such as a paucity in initiation events or an acceleration of replication forks.

DNA fiber analysis is also instrumental to detect spontaneous fork pausing events at a global level by measuring differences in the progression of sister replication forks. Indeed, when two unchallenged forks progressing from a given replication origin are labeled with successive pulses of IdU and CldU, the length of labeled tracks should be nearly identical. In contrast, pausing or stalling of one of the forks should result in an asymmetric pattern (Conti et al., 2007; Tuduri et al., 2009). Differences between the length of adjacent IdU and CldU tracks generated by a given fork is also indicative of increased pausing or stalling (Conti et al., 2007; Técher et al., 2013; Quinet et al., 2017).

Mild RS, defined as a reduction of fork rate lower than 50% compared to untreated cells, is well tolerated by mammalian cells. For instance, low doses of APH or HU do not activate the ATR-CHK1 pathway and do not induce detectable levels of DNA breaks even though they induce a significant slowdown of fork velocity (Bergoglio et al., 2013; Koundrioukoff et al., 2013; Toledo et al., 2013; Wilhelm et al., 2014; Saxena et al., 2018). This tolerance to mild RS conditions is due to the coordinated action of RS response pathways that stabilize, assist and restart paused forks (Toledo et al., 2017; Bellelli and Boulton, 2021). Tolerance to RS also depends on dormant replication origins (Figure 1B), which are present in large excess on chromosomes and act as backup to rescue stalled or collapsed forks (Técher et al., 2017).

In untreated cells, the rate of fork progression inversely correlates with the density of active origins (Anglana et al., 2003; Woodward et al., 2006; Ge et al., 2007; Courbet et al., 2008). Under low to mild HU or APH treatment, cells also activate dormant origins to compensate for slower forks (Ge et al., 2007; Courbet et al., 2008; Técher et al., 2016). This regulation operates within replication foci, which correspond to clusters of replication origins that are activated in a coordinated manner (Jackson and Pombo, 1998; Anglana et al., 2003; Conti et al., 2007). Within these replication foci, initiation events are distributed every 100–150 kb on average. When forks slow down, inter-origin distances (IODs) decrease due to the passive activation of dormant origins and via an active process mediated by ATR (Shechter et al., 2004; Lossaint et al., 2013; Chen et al., 2015). In response to low levels of RS (e.g., low dose of HU) the activation of dormant origins is promoted by FANCI and inhibited by FANCD2, likely through the phosphorylation of MCMs by the CDC7 kinase (Chen et al., 2015). The lack of ATR activation under low RS conditions is thus compatible with the firing of extra origins (Koundrioukoff et al., 2013). In contrast, initiation is repressed in response to high levels of RS (e.g., high doses of HU; Costanzo et al., 2003; Shechter et al., 2004; Ge and Blow, 2010). Under these conditions, ATR phosphorylates also FANCI, which then loses its ability to stimulate origin firing but promotes fork stability and restart together with FANCD2 (Lossaint et al., 2013; Chen et al., 2015; Figure 1B).

The ability to activate dormant origins near paused or arrested forks represents a strategy of choice to resume replication. Indeed, the excess of MCMs loaded onto chromatin is critical to maintain the availability of dormant origins in cancer cell lines. Under RS conditions, the partial depletion of MCMs abrogates the capacity of these cells to mobilize extra-origins and leads to increased chromosomal instability and cell death (Ge et al., 2007; Ibarra et al., 2008). In contrast, non-transformed cells are less dependent on high MCM levels to tolerate fork slowing.

Although this RS tolerance mechanism is very robust, it has some inherent limitations. The deleterious consequences of massive fork arrest or destabilization cannot be compensated by the firing of dormant origins and can even promote genome instability. Indeed, the activation of a large number of extra origins stresses the system by exhausting limiting factors. Work from the Lukas and Debatisse laboratories has shown that high levels of RS resulting from the combination of APH and ATR inhibition leads to replication catastrophe (Koundrioukoff et al., 2013; Toledo et al., 2013). In this context, the single-stranded (ss) DNA binding factor RPA becomes limiting, impacting both fork stability and checkpoint activation (Toledo et al., 2013). Moreover, activation of additional origins on a damaged template increases the number of stalled forks and generates additional substrates for structure-specific nucleases, contributing therefore to genomic instability (Pasero and Vindigni, 2017). In addition, dNTPs become limiting when too many origins are activated simultaneously in yeast and in mammalian cells (Petermann et al., 2010b; Mantiero et al., 2011).

Interestingly, dNTP levels also drop to suboptimal levels when budding yeast cells enter S phase and activate early replication origins. This dNTP shortage interferes with fork progression and activates the Mec1^*ATR*^ kinase, leading to the upregulation of RNR and to replication resumption (Forey et al., 2020). This transient RS represents therefore the physiological signal to coordinate the production of dNTPs with the onset of S phase. Finally, ATR is also important to couple S phase completion with mitosis onset during normal growth conditions in human cells. ATR inhibition or depletion results in premature entry into mitosis with genomic loci not being fully replicated. Under-replication leads to mitotic aberrations such as anaphase bridges and formation of chromosomal breaks (Eykelenboom et al., 2013; Saldivar et al., 2018).

In addition to the initiation rate, RS can also modulate fork speed. Somyajit and colleagues have recently shown that fork slowing in HU-treated cells depends on the sensing of oxidative stress by replisome components such as Timeless (Somyajit et al., 2017). Moreover, the ATR-CHK1 pathway actively reduces fork speed in response to RS. In human primary dermal fibroblasts exposed to HU, ATR slows down forks by targeting the MCM complex in a FANCD2-dependent manner (Lossaint et al., 2013). When forks face discrete impediments such as those caused by inter-strand crosslinks (ICLs), ATR signaling can also downregulate distant forks, although to a lesser extent than ICLs themselves (Mutreja et al., 2018). In budding yeast, replication forks progress faster when cells exposed to MMS or to low levels of DSBs are unable to activate Rad53^*CHK*1^, indicating that the DNA damage response actively reduces elongation (Bacal et al., 2018). Global fork slowing also relies on the CHK1 kinase in human cells exposed to low doses of the topoisomerase I inhibitor camptothecin (CPT) (Seiler et al., 2007). In CHK1-depleted cells, DNA lesions indirectly slow fork progression through the activation of the DNA damage response, notably via the ATM-p53 axis (Técher et al., 2016). In this latter case, fork slowing has been proposed to be, at least in part, the consequence dNTP starvation because the pool of dNTP has to be shared between repair and replication events (Técher et al., 2016).

## Pathological Consequences of Replication Stress

The RS response is activated in a variety of physiological and pathological situations. This chapter focuses on RS conditions that promote genomic instability and addresses how RS can be exploited in cancer treatment as a mean to overload tumor cells with an unbearable amount of DNA lesions (O’Connor, 2015). The pathological situations triggering an acute RS response differ significantly from the milder RS situations described above, in which functional checkpoint and repair pathways promote tolerance to low levels of RS. Acute RS situations are typically observed in cells defective for homologous recombination (HR) and ATR-CHK1 pathways, which accumulate RS and DNA damage markers (Kolinjivadi et al., 2017a; Técher et al., 2017). Hereafter, specific examples are discussed in which failure in one of these key pathways unveil their essential function in genome maintenance under RS conditions.

### Single-Stranded DNA Gaps at Stalled Forks

The RAD51 recombinase is a key HR factor that binds protruding 3′ single-stranded DNA (ssDNA) ends formed at resected double-strand breaks (DSBs) to form RAD51 filaments (Jasin and Rothstein, 2013). Resection of DNA ends is initiated by the endonuclease and 3′–5′ exonuclease MRE11 (Shibata et al., 2014) and is further extended by the long-range resection nucleases DNA2 and EXO1 (Jasin and Rothstein, 2013; Pasero and Vindigni, 2017). The key function of the RAD51 filament is to invade a donor DNA duplex harboring sequence homology to serve as template for DNA repair synthesis.

Pioneering work from Costanzo and colleagues using an electron microscopy (EM) approach developed by Jose Sogo (Sogo et al., 2002; Vindigni and Lopes, 2017) revealed that inactivation of RAD51 in *Xenopus* egg extract leads to the formation of ssDNA gaps at arrested forks (Hashimoto et al., 2010). Together with studies from the Jasin group (Schlacher et al., 2011, 2012), these seminal observations unveiled a novel role for HR factors in the protection of nascent DNA at stalled forks. In this process, RAD51 is loaded on newly replicated chromatin by BRCA1 and BRCA2, as shown by iPOND (isolation of proteins on nascent DNA) and related assays (Petermann et al., 2010a; Zellweger et al., 2015; Kolinjivadi et al., 2017b), to prevent the excessive degradation of nascent DNA strands by MRE11 (Hashimoto et al., 2010; Schlacher et al., 2011). The fork protection mechanism mediated by RAD51 depends on its ability to form a fully functional nucleofilament (Zadorozhny et al., 2017).

Fork resection is a conserved mechanism that has been reported in different species, from yeast to *Xenopus*, mice and human (Schlacher et al., 2011, 2012; Somyajit et al., 2015; Ait Saada et al., 2017; Kolinjivadi et al., 2017b; Lemacon et al., 2017; Mijic et al., 2017; Delamarre et al., 2020). Importantly, fork resection is not a pathological process *per se*, but rather a physiological event promoting HR-mediated fork repair and contributing to the activation of the ATR-CHK1 pathway (Coquel et al., 2018; García-Rodríguez et al., 2018; Menin et al., 2018; Villa et al., 2018; Delamarre et al., 2020). Accordingly, fork resection has been reported in a variety of HU-treated human cells, including U2OS, HeLa, and HEK293T cells (Thangavel et al., 2015; Bhat et al., 2018; Coquel et al., 2018). However, nascent DNA resection must be tightly controlled to prevent irreversible fork collapse.

**FIGURE 2:**
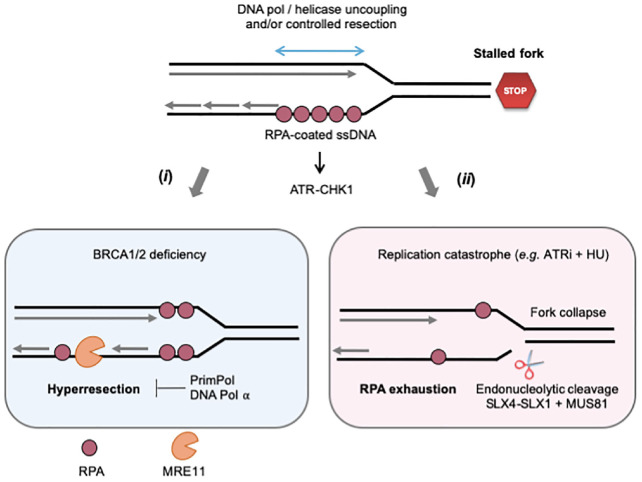
Controlled and pathological formation of ssDNA at stalled forks. When cells are exposed to RS, ssDNA forms at stalled forks through the uncoupling of replicative helicase and polymerase activities and/or the controlled degradation of nascent DNA by MRE11 and other nucleases. The ssDNA exposed is covered by the ssDNA-binding protein RPA, which serves as platform to recruit and activate ATR-CHK1 signaling. (*i*) In BRCA2-deficient cells, large ssDNA gaps form as the result of uncontrolled MRE11-mediated nascent DNA degradation (hyperresection). DNA pol α and PrimPol could either fill these gaps or promote lesion bypass while creating additional gaps in the process. (*ii*) The ATR-CHK1 pathway represses initiation when cells are exposed to acute RS conditions (high doses of APH or HU). In the presence of ATRi, the increased number of stalled forks depletes RPA and cells suffer from replication catastrophe, an event characterized by increased ssDNA exposure and massive fork collapse. Fork breakage may also result from uncontrolled nuclease activities, such as MUS81, SLX4-1. See text for further details.

The nature of MRE11 substrates at stalled forks is currently the subject of intense research (Figure 2). In RAD51-deficient *Xenopus* egg extracts, ssDNA gaps are detected both immediately behind stalled forks and at internal sites on daughter strands (Hashimoto et al., 2010). Only internal gaps depend on MRE11, the ssDNA gaps observed at the fork junction potentially resulting from an uncoupling between DNA polymerase and helicase activities (Zou and Elledge, 2003; Byun et al., 2005). Interestingly, most internal gaps show an asymmetric distribution on daughter strands (Hashimoto et al., 2010; Kolinjivadi et al., 2017b). An attractive possibility could be that incomplete Okazaki fragment processing on the lagging strand provides entry points for MRE11-mediated degradation. This view is supported by the fact that RAD51 interacts with DNA polymerase alpha (Pol α) and that RAD51 depletion leads to a decreased loading of Pol α at forks, leading to incomplete Okazaki fragments synthesis (Kolinjivadi et al., 2017a,b). Moreover, inhibition of Pol α with the small-molecule inhibitor CD437 interferes with lagging strand synthesis and also leads to the accumulation of ssDNA gaps on one of the daughter strands (Ercilla et al., 2020). Although current EM techniques cannot discriminate between leading and lagging strands, these data strongly suggest that MRE11 acts on the lagging strand to generate ssDNA gaps in *Xenopus* egg extracts. In other organisms, it has been proposed that MRE11 can also act by enlarging ssDNA gaps generated upon repriming of DNA synthesis after a lesion on the leading strand (García-Rodríguez et al., 2018; Quinet et al., 2020; Somyajit et al., 2021). As it is the case for DSB end resection, the exonuclease activity of MRE11 is stimulated by its interaction with CtIP and SAMHD1 (Daddacha et al., 2017; Coquel et al., 2018). Moreover, long-range resection at stalled forks involves additional nucleases and DNA helicases such as EXO1 and BLM (Berti and Vindigni, 2016; Pasero and Vindigni, 2017).

### Fork Reversal as a Mechanism to Protect and Restart Arrested Forks

**FIGURE 3:**
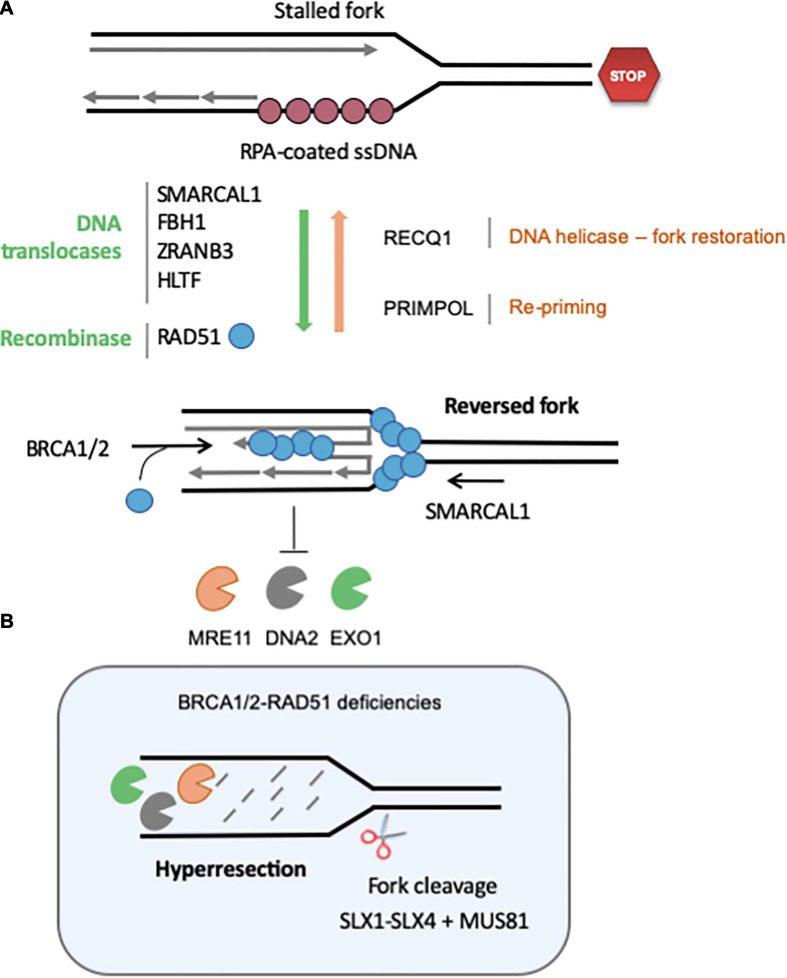
Regulation of the formation and stability of reversed fork. **(A)** Fork reversal results from the re-annealing of parental strands, zipping the fork backward and promoting the pairing of nascent strands. Fork reversal is highly regulated, and many factors are currently known to regulate the balance between RVF formation, stabilization and restart. Redundant DNA translocases, namely SMARCAL1, FBH1, ZRANB3, and HLTF, have been described to promote fork regression *in vitro* and *in vivo*. The recombinase RAD51 also promotes fork reversion in a BRCA2-independent manner, although it is not clear whether its strand invasion property is required. RECQ1 is the major helicase known to promote restoration of the normal fork structure. PARP1 inhibits RECQ1-mediated fork restoration. Re-priming of DNA synthesis through PrimPol favors fork restart and reduces the frequency of fork regression. Once formed, RVF are stabilized by BRCA2-mediated loading of RAD51 on the regressed arm. **(B)** If BRCA-RAD51 fork protection is not functional, the regressed arm is extensively degraded by MRE11, DNA2 and EXO1. Eventually, the SLX1-SLX4-MUS81 endonucleases can cleave these structures to rescue forks. See text for further details.

Reversed forks (RVFs) result from the extensive remodeling of stalled forks into branched structures resembling Holliday junctions (HJs). HJs are formed during HR by strand invasion of a homologous template in a RAD51-dependent manner. Similarly, reversed forks (RVFs) result from the reannealing of parental DNA strands, zipping the fork backwards and allowing the concomitant pairing of newly synthesized strands (Figure 3A). RVFs were first visualized in budding yeast by EM, using psoralen-crosslinked DNA samples to prevent branch migration after DNA extraction (Sogo et al., 2002). EM analysis remains the gold standard to monitor RVF formation and stability in large vertebrate genomes (Vindigni and Lopes, 2017).

RVFs were initially observed in HU-treated yeast mutants deficient for the checkpoint kinase Rad53^*CHK*1^, but not in wild-type cells (Sogo et al., 2002). This led to the assumption that RVFs are pathological structures corresponding to terminally arrested forks. However, several lines of evidence indicate that fork reversal is rather an active process contributing to the protection and the repair of arrested forks through an HR-mediated process that does not require the formation of a DSB (Berti and Vindigni, 2016; Giannattasio and Branzei, 2017; Teixeira-Silva et al., 2017). This raises important questions regarding (*i*) the conditions under which fork reverse, (*ii*) the factors that regulate fork reversal, (*iii*) the consequences of fork reversal in normal and pathological situations, and (*iv*) the requirement of a fork reversal for resection.

### Conditions That Promote Fork Reversal

RVFs are rarely detected in unchallenged conditions (Sogo et al., 2002; Ray Chaudhuri et al., 2012; Zellweger et al., 2015; Kolinjivadi et al., 2017a), indicating either that fork reversal does not occur at natural pause sites and spontaneous DNA lesions or is too transient to be detected by EM. The frequency of RVFs increases dramatically when yeast cells and Xenopus egg extracts are exposed to low doses of the topoisomerase I inhibitor CPT (Ray Chaudhuri et al., 2012; Menin et al., 2018). Since topoisomerases release positive DNA supercoiling accumulating ahead of the replication fork, these data suggest that DNA torsional stress contributes to fork reversal. However, RVFs are also frequently detected in human cell lines exposed to a large panel of DNA damaging agents that do not necessarily accumulate DNA supercoiling (Zellweger et al., 2015). For instance, 20–30% of the replication intermediates (RIs) detected under mild RS conditions (40–60% fork slowdown) correspond to RVFs (Zellweger et al., 2015). These results suggest that fork reversal may significantly contribute to reduce fork rates. They also indicate that besides torsional stress, cellular factors may actively promote fork reversal. Fork reversal may also constitute a structure prone to recruit repair factors and allowing access to DNA damage on the template (Neelsen and Lopes, 2015; Berti and Vindigni, 2016).

### Factors Regulating Fork Reversal

The DNA translocase SMARCAL1 is one of the first protein shown to cause fork reversal *in vivo* and *in vitro* (Bétous et al., 2012; Couch et al., 2013). This factor is responsible for half of RVFs in APH-treated *Xenopus* egg extract (Kolinjivadi et al., 2017b) and it plays a predominant role in fork reversal in BRCA1/2-deficient mammalian cells (Taglialatela et al., 2017). Other DNA translocases such as FBH1, HLTF, and ZRANB3 also play roles in the formation of RVFs, depending on the cell-type and the drugs used to induce RS (Fugger et al., 2015; Mijic et al., 2017; Vujanovic et al., 2017; Bai et al., 2020). It has been proposed that SNF2-family of DNA translocases may act hierarchically, SMARCAL1 and HLTF being first recruited on RPA-coated ssDNA to initiate fork reversal. In a second step, HLTF would recruit ZRANB3 through poly-ubiquitylation of PCNA to extend fork reversal (Taglialatela et al., 2017; Vujanovic et al., 2017; Bai et al., 2020). Further extension of RVFs downstream of SNF2-fork remodelers is mediated by topoisomerase II and the helicase PICH (Tian et al., 2021). In topoisomerase II- or PICH-depleted cells, reversed forks are less frequent and they show shorter regressed arms, suggesting that they are less stable. These translocases may also act on different substrates or fork structures, which would explain this apparent functional redundancy. It has been recently shown that 53BP1 protects forks that are reversed by FBH1 in U2OS cells (Liu et al., 2020), whereas BRCA2 protects SNF2-remodeled forks. These results point to the existence of several mechanisms of fork reversion and stabilization.

ATR phosphorylates SMARCAL1 in response to RS (Couch et al., 2013) and could therefore regulate fork reversal. It has been shown that ATR inhibits SMARCAL1 activity *in vitro* and, as a consequence, it has been proposed that ATR activity restrains fork reversal. This hypothesis is supported by the finding that more ssDNA is exposed in ATR-inhibited cells in a SMARCAL1-dependent manner (Couch et al., 2013). However, this view is not consistent with recent EM studies from Lopes and co-workers, showing that ATR inhibition abrogates fork reversal under diverse RS conditions (Mutreja et al., 2018). However, ATR inhibition has pleiotropic effects and could affect fork reversal indirectly, for instance by interfering with the localization or the function of HR factors such as RAD51 (Sorensen et al., 2005; Buisson et al., 2017).

RAD51 is another key regulator of fork reversal. Indeed, the partial depletion of RAD51 decreases the frequency of RVFs in human cells (Zellweger et al., 2015) and in *Xenopus* egg extracts (Kolinjivadi et al., 2017b). Moreover, SMARCAL1 and RAD51 depletion have additive effects on fork regression, showing that their mechanism of action is different. Interestingly, this function of RAD51 is also distinct from its role in the protection of nascent DNA against MRE11-dependent degradation. Since MRE11 acts on reversed forks, RAD51 could therefore promote fork resection both by contributing to fork reversal in a BRCA2-independent manner and by protecting nascent DNA from degradation in a BRCA2-dependent manner (Kolinjivadi et al., 2017b; Lemacon et al., 2017; Mijic et al., 2017).

RVFs can also form when DNA Pol α function is compromised in yeast primase mutants (Fumasoni et al., 2015) and in the absence of TIM (Timeless) and Tipin (Errico et al., 2007, 2014), two components of the fork protection complex (FPC). Since TIM and Tipin promote the recruitment of Pol α to chromatin (Errico et al., 2014), this suggests the existence of a link between RVFs and defects in Pol α-dependent DNA synthesis. Importantly, both Pol α and the FPC prevent the accumulation of ssDNA gaps at forks. Indeed, Pol α is required for lagging strand synthesis and the FPC coordinates the activity of DNA polymerases and helicases (Katou et al., 2003; Errico et al., 2009; Kurat et al., 2017; Abe et al., 2018). In addition, Pol α may reprime DNA synthesis at stalled forks and contribute to the filling of post-replicative ssDNA gaps, presumably through the interaction between Pol α and RAD51 (Kolinjivadi et al., 2017b). This balance between resection and repriming is well documented at unprotected telomeres, where EXO1-mediated resection is compensated by Pol α gap filling (Wu et al., 2012) and could also occurs during DSB repair (Mirman et al., 2018). Moreover, the Pol α interactor AND1 is important to promote both fork progression and protection (Abe et al., 2018). Increased fork reversal upon AND1 or Tipin depletion suggests that ssDNA gaps promote RVFs (Errico et al., 2014; Abe et al., 2018), as illustrated by the presence of single-stranded tails at RVFs (Thangavel et al., 2015; Kolinjivadi et al., 2017b; Lemacon et al., 2017).

Another important regulator of fork reversal is the poly-ADP-ribose polymerase 1 (PARP1). In particular, PARP1 promotes RVF in response to topoisomerase I inhibition by CPT (Ray Chaudhuri et al., 2012; Berti et al., 2013), but the mechanism involved remains elusive. PARP1 interacts with RECQ1, a DNA helicase involved in the resolution of RVFs (Berti et al., 2013) and PARP1 inhibition reduces the frequency of CPT-induced RVFs in controls cells but not in RECQ1-depleted cells. RECQ1 could therefore be the main target of PARP1 to stabilize RVFs by preventing their resolution by RECQ1.

The expression of p53 has been shown to restrain fork progression and to promote recombination events in absence or presence of exogenous RS inducers (Hampp et al., 2016; Biber et al., 2021). P53 interacts with the translesion synthesis polymerase ι (POLι) and PCNA that promote a mechanism called “idling,” which acts as a replication barrier and gives time for HLTF to poly-ubiquitylate PCNA. The DNA binding and interaction with RPA of p53 are required for this DNA damage tolerance pathway, but transcription regulation is not (Biber et al., 2021). It has been suggested that this “idling” mechanism promotes fork reversal by the combined action of HLTF and ZRANB3, leading to the observed fork deceleration and increase frequency of recombinational events during S phase.

PrimPol, a DNA polymerase with primase activity, has recently been discovered and acts in a parallel pathway to fork reversal (Bianchi et al., 2013; Mourón et al., 2013; Wan et al., 2013). PrimPol promotes fork progression under RS conditions induced by UV irradiation or HU treatment by repriming DNA synthesis at stalled forks. PrimPol defects lead to the persistence of ssDNA gaps (Vallerga et al., 2015). In the absence of RAD51, cells accumulate ssDNA gaps in a PrimPol dependent-manner, indicating that PrimPol promotes constant re-priming in these cells, at the expense of nascent DNA integrity (Vallerga et al., 2015). Moreover, PrimPol restrains nascent DNA degradation in BRCA-deficient cells by preventing fork reversal (Quinet et al., 2020). Conversely, inhibition of fork reversal by SMARCAL1 or HLTF depletion favors PrimPol-mediated repriming (Bai et al., 2020; Quinet et al., 2020). The balance between RAD51-SMARCAL1 and PrimPol dictates the choice between fork reversal and repriming, with potential consequences on the degradation of RVFs by MRE11 (i.e., in BRCA deficient background) or the persistence of ssDNA gaps.

Importantly, the balance between fork reversal and repriming also impacts on the speed of replication forks, as measured by DNA fiber assays. Indeed, the fork slowdown induced by MMC or CPT in human cells depends on RAD51 (Zellweger et al., 2015) and the effect of Cisplatin and UV on chicken DT40 cells depends on the RAD51 paralog XRCC3 (Henry-Mowatt et al., 2003). Since XRCC3 regulates RAD51 activity, both factors could indirectly regulate fork speed by promoting fork reversal. Along the same line, the depletion of SMARCAL1 or PARP1 increases fork speed in a variety of contexts (Berti et al., 2013; Mijic et al., 2017; Vujanovic et al., 2017; Maya-Mendoza et al., 2018) and p53 expression leads to fork deceleration (Biber et al., 2021). Altogether, these studies show that the apparent speed of replication forks inversely correlates with the rate of fork reversal.

### Fork Reversal in Pathological Situations

A large body of evidence indicates that the controlled resection of nascent DNA by nucleases such as MRE11, EXO1 and DNA2 contributes to the recovery of stalled forks (Trenz et al., 2006; Thangavel et al., 2015). However, this control is lost in the absence of BRCA1/2 and hyper-resection leads to fork collapse and chromosome breaks (Schlacher et al., 2011, 2012; Wilhelm et al., 2014; Feng and Jasin, 2017; Taglialatela et al., 2017; Figure 3B). Hyper-resection of nascent DNA in BRCA-deficient cells generates structures that need to be cleaved by MUS81 to resume replication (Lemacon et al., 2017). SMARCAL1 causes genomic instability in BRCA1-deficient cells by promoting the formation of large ssDNA gaps (>300 nt) at stalled forks in a MRE11-dependent manner, which may in turn generate ultrafine chromatin bridges in mitosis (Vujanovic et al., 2017). In BRCA1/2-deficient cells, SMARCAL1 depletion restores replication fork stability and reduces the formation of replication stress-induced DNA breaks and chromosomal aberrations (Taglialatela et al., 2017).

Fork resection depends on PTIP, a protein interacting with members of a family of histone H3K4 methyltransferases known as MLL (Mixed Lineage Leukemia). PTIP promotes MRE11 and RAD51 loading to chromatin at the level of nascent DNA. In BRCA1/2-deficient cells, PTIP knock-out (KO) alleviates hyper-resection of nascent DNA and chromosome breaks (Ray Chaudhuri et al., 2016). These results suggest that chromatin modifications may influence fork stability, presumably through the recruitment of nucleases. Moreover, it has been recently shown that the lysine acetyltransferase KAT2B (also known as PCAF) that acetylates core histones promotes nascent DNA degradation in BRCA-deficient cells. PCAF acetylates H4 at lysine 8 at the level of stalled forks, promoting the recruitment of the MRE11 and EXO1 nucleases (Kim et al., 2020). BRCA2-deficient tumor cells have been shown to resist to cisplatin treatment in the absence of the nucleosome remodeler CHD4 (part of NuRD complex) through an increase level of translesion synthesis (Guillemette et al., 2015). Cisplatin resistance in this latter context has not been correlated to a particular state of chromatin but it has been shown that CHD4 loss restores fork stability in BRCA2-deficient cells (Ray Chaudhuri et al., 2016).

Nascent DNA degradation also depends on PARP1 status. As mentioned earlier, PARP1 inhibition decreases the level of RVFs and blocks fork resection in BRCA-deficient cells (Ray Chaudhuri et al., 2012, 2016; Ding et al., 2016). Either PARP inhibition or PARP1 depletion has been shown to promote the viability of BRCA1-2 deficient cells, especially in the context of ESCs (Embryonic Stem Cells) (Ding et al., 2016). Along the same line, MRE11 depletion or inhibition with Mirin treatment restores the viability of BRCA2^–/–^ ESCs (Ray Chaudhuri et al., 2016). Together, these data indicate that the lethality of ESCs caused by BRCA1-2 deficiency stems from the degradation of nascent strands at RVFs in a mechanism dependent on PARP1 and MRE11. These results contrast with the known sensitivity of BRCA-deficient cancer cells to PARPi. In these BRCA-deficient cancer cells, survival depends on PARP-mediated DNA repair as an alternative to HR.

In conclusion, a large body of evidence supports the view that fork reversal is a physiological process protecting replication forks against exogenous sources of RS. Recently, this role was extended to oncogene induced-RS. Indeed, both SMARCAL1 and ZRANB3 protect cells against Myc-induced RS (Puccetti et al., 2019) and behave therefore as tumor suppressors. Preventing fork reversal through the chemical inhibition of SMARCAL1 or ZRANB3 could provide a new line of anticancer treatment. This strategy would be especially relevant in combination to drugs that induce RS or in genetic backgrounds associated with fork instability.

## Replication Stress Induces Fork Cleavage and Mitotic Defects

Although cells generally manage to complete S phase under mild RS conditions, they usually display increased levels of mitotic aberrations and chromosomal abnormalities, such as metaphase breaks or anaphase bridges. Moderate RS levels also increase chromosome breaks at CFS (Debatisse et al., 2012), at least in part through the cleavage of late-replication intermediates by the ERCC1 and MUS81-EME1 nucleases (Naim et al., 2013; Ying et al., 2013) and by preventing the complete duplication of these loci until the onset of mitosis. Interestingly, depletion of MUS81-EME1 in aphidicolin-treated cells reduces chromosomal breaks at the expense of a sharp increase of anaphase bridges. These data indicate that the MUS81 and ERCC1 cleave under-replicated DNA in mitosis to avoid more deleterious consequences associated with the persistence of entangled chromosomes. Indeed, chromatin bridges have a high likelihood to break during anaphase, resulting in the loss of genomic DNA and the formation of micronuclei. It has been shown that MUS81 cleavage at CFSs triggers a mitotic mechanism of DNA synthesis and repair involving POLD3 (Minocherhomji et al., 2015). This mitotic DNA synthesis (MIDAS) represents the last opportunity to complete the duplication of under-replicated CFSs, presumably through the cleavage of RVFs in G_2_/M. Fugger et al. have shown that RVFs are formed by FBH1 and then cleaved by MUS81, forming DSBs (Fugger et al., 2013, 2015), suggesting that RVFs are a good substrate for structure-specific nucleases and are cleaved to promote fork recovery. This is consistent with the fact that MUS81 promotes cell viability in the presence of APH or HU by inducing DSBs (Hanada et al., 2007). Failure to resolve these late intermediates leads to the formation of 53BP1 bodies in the next cell cycle (Lukas et al., 2011).

HR deficiency leads to the accumulation of bulky anaphase bridges, reflecting the persistence of repair intermediates or under-replicated DNA in mitosis (Wilhelm et al., 2014; Ait Saada et al., 2017; Feng and Jasin, 2017; Lai et al., 2017). In BRCA-deficient cells, this phenotype is further increased by the depletion of MUS81 (Lai et al., 2017), which is reminiscent of CFS breakage under mild RS condition. In budding yeast, survival to CPT relies on both HR factors, namely Rad51 and Rad52, and the structure specific Mus81 endonuclease (Pardo et al., 2020). In both *rad51* and *rad52* mutants, replication tracks are shorter upon CPT-treatment, indicating that the formation of recombination structures (i.e., D-loop) protects arrested forks. In this context, MUS81 is important to resolve intermediates in G_2_/M phase of the cell cycle. The fact that Mus81 acts in G_2_/M in response to CPT suggests that the stalled fork, after being protected through Rad52-Rad51-mediated strand invasion, is joined by a converging fork, enabling completion of the replication after Mus81 cleavage. This model is consistent with the fact that late replication intermediates at human CFSs are also resolved, at least in part, by MUS81 in G_2_/M (Naim et al., 2013; Ying et al., 2013). Replication forks are slower in BRCA2- and RAD51-deficient cells (Wilhelm et al., 2014, 2016; Lai et al., 2017), but restoration of normal fork progression by supplementing cells with nucleotide precursors and anti-oxidants also abolishes mitotic defects (Wilhelm et al., 2014, 2016). These results show that fork slowing in HR-deficient cells mimics low RS and leads to incomplete DNA replication and mitotic aberrations.

As discussed above, the controlled cleavage of RVFs and other replication intermediates promotes tolerance to RS. However in some instances uncontrolled cleavage of DNA by nucleases leads to the accumulation of high load of DNA breaks that is detrimental to cells (Pasero and Vindigni, 2017; Figures 2, 3). This is the case when S-phase checkpoints are not fully functional. For instance, CHK1 deficiency leads to the accumulation of DNA lesions formed by MUS81-EME2 and MRE11 (Syljuasen et al., 2005; Técher et al., 2016; Calzetta et al., 2020). This uncontrolled cleavage may reflect the unscheduled activation of CDKs in the absence of CHK1, which is known to regulate both MUS81 and MRE11 activities. Consistently, inhibition of CDK by Roscovitine abolishes the appearance of DNA damages in CHK1-deficient cells (Syljuasen et al., 2005).

## Self DNA and Activation of the Inflammatory Response

Cancer cells bear a high load of chromosomal instability and suffer from replication defects, which both induce inflammation. This inflammation arises from the pathological accumulation of genomic DNA fragments in the cytoplasm when chromosome integrity is compromised. The pioneering work of Nelson Gekara’s group revealed that genomic instability correlates with induction of inflammation (Härtlova et al., 2015). These authors found that ATM deficiency and DNA damage inducers such as γ-irradiation and Etoposide, promote the accumulation of cytosolic DNA molecules and trigger a type I interferon (IFN) response. However, the nature and the origin of cytosolic DNA species remained poorly understood.

Early studies from Costanzo and colleagues have shown that in *Xenopus* egg extracts, the processing of DSBs by the MRE11 nuclease leads to the release of short oligonucleotides from DNA ends, which contribute to activate ATM (Jazayeri et al., 2008). Irradiation of human cells also leads to the release of soluble ssDNA oligos (Jazayeri et al., 2008). More recently, a variety of agents used in radiotherapy and chemotherapy were shown to promote the accumulation of ssDNA fragments in the cytosol and the activation of a type I interferon response (Erdal et al., 2017). The impact of this DNA damage-induced inflammatory response has broad consequences for cancer therapy, as acute inflammation promotes tumor rejection by the immune system (Erdal et al., 2017; Harding et al., 2017; Mackenzie et al., 2017). However, chronic inflammation associated with chromosomal instability in some cancer types may also promote cancer development by stimulating metastasis (Bakhoum et al., 2018), stressing the importance of better understanding the links between RS and inflammation.

### The Aicardi-Goutières Syndrome

Recent advances in the analysis of genes frequently mutated in the Aicardi-Goutières syndrome (AGS) has shed new light into the molecular mechanisms linking RS and inflammation. AGS is a severe interferonopathy associated with microcephaly and chronic inflammation (Crow and Manel, 2015). Cells from AGS patients accumulate cytosolic nucleic acids and show a chronic induction of type I IFNs via the cGAS (cyclic GMP-AMP synthase)-STING (stimulator of interferon genes) pathway. cGAS is a DNA sensor producing cyclic GMP-AMP (cGAMP) as a second messenger upon binding to dsDNA and activating the transcription of interferon genes via the STING-TBK1-IRF3 axis (Li and Chen, 2018).

The mechanism by which AGS cells accumulate self DNA in their cytoplasm has long remained elusive. However, recent evidence indicates that byproducts of nuclear DNA repair and/or replication fork processing could represent a major source of cytosolic DNA in AGS cells (Erdal et al., 2017; Mackenzie et al., 2017; Yang et al., 2007; Coquel et al., 2018). This is consistent with the genetics of AGS, involving several enzymes processing nucleic acids, such as the cytoplasmic exonuclease TREX1, the ribonuclease RNase H2 or the dNTPase SAMHD1 (Crow and Manel, 2015).

TREX1 degrades cytosolic DNA species (Yang et al., 2007; Stetson et al., 2008) and has been proposed to process “abnormal” DNA structures at hard-to-replicate telomeres (Maciejowski et al., 2015), even though this role was challenged by a more recent study (Umbreit et al., 2020). The ssDNA binding factors RPA and RAD51 protect TREX1-deficient cells from inflammation by sequestering DNA fragments in the nucleus (Wolf et al., 2016; Vanpouille-Box et al., 2017). These results are consistent with the hypothesis that byproducts of DNA repair reactions induce inflammatory signals in AGS cells.

SAMHD1 mutations are implicated in several human diseases among which AGS (Rice et al., 2009), viral infection and cancers (Crow and Manel, 2015). SAMHD1 is a dNTP triphosphohydrolase (dNTPase) that degrades dNTPs and enables cells to control dNTP pools level and balance (Franzolin et al., 2013). In addition to dNTP control, SAMHD1 also impacts replication and repair fidelity through its DNA binding and interaction with the CtIP-MRE11 nuclease (Seamon et al., 2015; Daddacha et al., 2017; Coquel et al., 2018). SAMHD1-deficient cells accumulate cytosolic DNA fragments in response to RS (Coquel et al., 2018).

RNase H2 is a three-subunit enzyme involved in the removal of different types of RNA:DNA hybrids. Mutations in RNase H2 subunit are associated with AGS (Crow et al., 2006) and with chromosomal instability (Mackenzie et al., 2017). Deletion of RNase H2 genes is embryonic lethal in mouse (Reijns et al., 2012). RNase H2 removes ribonucleotides misincorporated into DNA (Reijns et al., 2012; Sparks et al., 2012) and processes R-loops (Lim et al., 2015). It has been recently proposed that micronuclei formation in absence of RNase H2 is the source of pro-inflammatory self DNA (Mackenzie et al., 2017).

### Origin of Cytosolic DNA in Cancer Cells

RS and DNA damage induce the accumulation of cytosolic DNA, especially in TREX1-deficent cells (Yang et al., 2007; Stetson et al., 2008). But how are these chromosomal DNA fragments released from the nucleus? Two main mechanisms have been described in response to genotoxic insults. On the one hand, incomplete DNA replication or defective DSB repair generate large chromosome fragments devoid of centromeres that form micronuclei after mitosis. In different contexts, including RNase H2 deficiency, γ-irradiation, Ras overexpression and BRCA2 mutation, the rupture of these micronuclei releases DNA fragments in the cytosol and activates the cGAS-STING pathway (Dou et al., 2017; Gluck et al., 2017; Mackenzie et al., 2017; Reisländer et al., 2019). On the other hand, small DNA fragments are directly released from DNA ends (Jazayeri et al., 2008; Erdal et al., 2017) and escape the nucleus (Wolf et al., 2016). In SAMHD1-deficient cells, nascent DNA is displaced from stalled forks as a consequence of aberrant fork processing by RECQ1 and MRE11 (Coquel et al., 2018). This is reminiscent of the BLM- and EXO1-dependent release of ssDNA from damaged DNA (Erdal et al., 2017) and indicates that alterations of classical resection pathways contribute to the release of DNA fragments from the nucleus.

A growing body of evidence indicates that stalled and reversed forks represent a major source of cytosolic DNA when cells are exposed to genotoxic agents interfering with DNA replication. Central to this process is the role of endonucleases that release DNA fragments from arrested forks. In prostate cancer cells, the structure-specific endonuclease MUS81 is necessary for the accumulation of cytosolic DNA fragments (Shen et al., 2015; Ho et al., 2016). Interestingly, this process depends also on PARP1 and ATR (Ho et al., 2016; Kidiyoor et al., 2020), which are involved in fork reversal and restart. However, the role of RVFs in the production of cytosolic DNA remains to be established.

**FIGURE 4:**
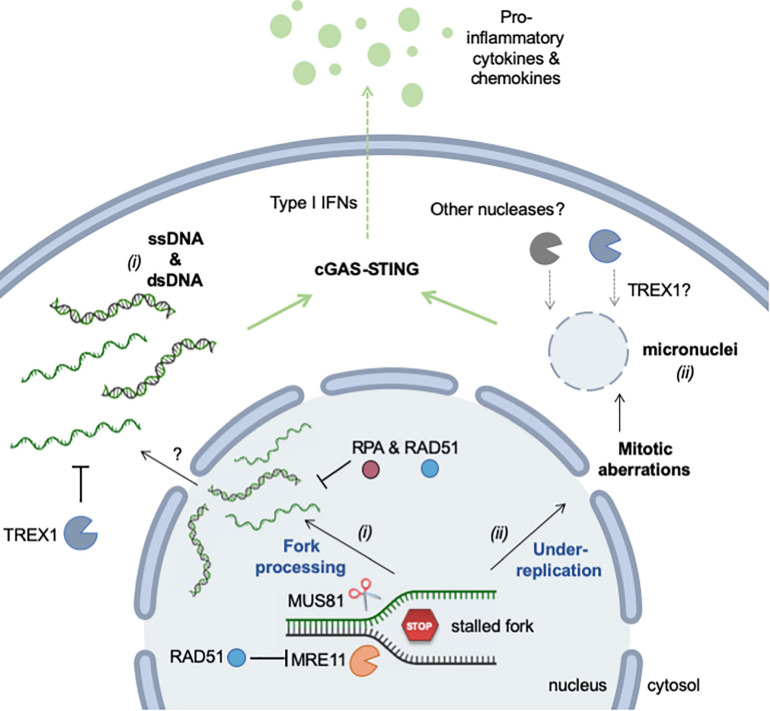
Mechanisms of RS-induced accumulation of cytosolic (self-) DNA. Self DNA accumulates in the cytosol when mammalian cells are exposed to RS-inducing agents (e.g., HU, APH, CPT) or when cells are deficient for factors promoting replication fork stability (e.g., SAMHD1). The release of genomic DNA occurs in two different ways. (*i*) DNA fragments (ssDNA and dsDNA) are released from stalled forks by nucleolytic cleavage (MRE11 and MUS81). The mechanism by which these fragments exit the nucleus is currently unknown, but it is repressed by RPA and RAD51. TREX1 is the major cytosolic exonuclease that degrades DNA to prevent the activation of the cGAS-STING pathway. (*ii*) Incomplete replication, known as under-replication, induces chromosome segregation defects and leads to the formation of micronuclei and to other mitotic defects. Upon rupture of their membrane, cGAS localizes to micronuclei and activates STING. Whether TREX1 or other nucleases can access DNA inside micronuclei is not known. See text for additional details. This figure was created in part with BioRender.com.

In conclusion, RS promotes the accumulation of cytosolic DNA via two distinct mechanisms: (*i*) the formation and rupture of micronuclei resulting from the missegregation of chromosomes during mitosis and (*ii*) the aberrant processing of stalled replication forks (Figure 4). These two mechanisms generate DNA fragments of different size and structure, but are not mutually exclusive. They may represent the two faces of the same coin, activating inflammation in response to different types of replication stress.

### Exploiting Replication Stress-Induced Inflammation in Cancer Treatment

Inflammation is a two-edged sword in the context of cancer treatment. Although inflammation induced by irradiation or chemotherapeutic agents contributes to tumor cell rejection (Erdal et al., 2017; Gluck et al., 2017; Mackenzie et al., 2017), chronic inflammation contributes to cancer development by promoting metastasis (Bakhoum et al., 2018). Exploiting RS-mediated inflammation to potentiate the effect of current cancer therapies requires therefore a thorough understanding of the molecular mechanisms involved.

Immunotherapy has made considerable progress during the past decade with the development of potent immune checkpoint inhibitors to unlock the immune rejection of cancer cells. However, these inhibitors are useless against “cold” tumors that escape detection by the immune system. Stimulating inflammation in these tumors in a controlled manner could represent a promising strategy to increase tumor infiltration by immune cells and potentiate the action of immune checkpoint inhibitors. Actionable targets to modulate this response include TREX1 and the STING pathway. Indeed, TREX1 is an upstream regulator of radiation-driven anti-tumor immunity (Vanpouille-Box et al., 2017) and STING is essential to promote tumor rejection in immunocompetent mice treated with the anticancer agent Topotecan (Kitai et al., 2017). The cGAS-STING pathway is also often deregulated in cancer cells, supporting the view that it interferes with tumor growth (Lau et al., 2015). Strategies targeting DNA integrity and the DNA damage response could also have additive or synergistic effects with immunotherapies. Thus, it has been recently shown that ERCC1-deficient non-small cell lung cancer (NSCLC) cells accumulate cytosolic chromatin fragments and consecutively induce type I IFNs in response to PARP inhibition (Chabanon et al., 2019). NSCLC cells treated with PARP inhibitors (PARPi) induce the cell surface expression of the PD-L1 immune checkpoint inhibitor and secrete the CCL5 chemokine (Chabanon et al., 2019). In patient tumor samples, ERCC1-deficiency is associated with increased levels of lymphocyte infiltration, indicating that the type I IFN response observed in cultured cells occurs *in vivo*, promoting the attraction of immune cells. This proof-of-concept was also recently established *in vivo* using an elegant preclinical model of small cell lung cancer (Sen et al., 2019). In these mice, anti-PD-L1, PARPi or CHK1 inhibitors (CHK1i) have only a modest effect on tumor growth when used alone. However, both PARPi and CHK1i have synergistic effects on tumor growth when administrated in combination with anti-PD-L1 immunotherapy in mice proficient for the cGAS-STING pathway. In STING- or cGAS-deficient mice, the combination of PARPi/antiPD-L1 or CHK1i/antiPD-L1 had no effect on tumor growth (Sen et al., 2019). These data show that under these circumstances, self-DNA sensing is essential to promote the immune rejection of cancer cells.

### Beyond cGAS DNA Sensing

It has been recently shown that cGAS localizes to the nucleus and is recruited to DNA damage foci, where it inhibits HR-mediated DNA repair (Liu et al., 2018; Jiang et al., 2019). Through its chromatin occupancy and DNA compaction, cGAS impedes RAD51 strand invasion during repair, impacting genome integrity and cell survival to DNA insults (Jiang et al., 2019). It is thus important to consider this STING-independent function of cGAS in particular when investigating the contribution of the cGAS-STING pathway to the response to chemotherapy.

Although DNA sensing by cGAS-STING plays a major role in the response to cancer therapy, as described above, it should be noted that RNA in various forms can contribute to the inflammatory response under conditions that challenge genome integrity. For instance the inhibition of ATR in irradiated cells induces type I IFNs by the RNA sensing pathway RIG-I/MDA5 (Feng et al., 2020). Moreover, TLR9 and its adaptor protein MyD88 or cGAS have been shown to detect RNA:DNA hybrids (Mankan et al., 2014; Rigby et al., 2014). R-loops have been extensively described as a potential source of fork impediment and DNA damage (Zeman and Cimprich, 2014). Yet, whether R-loop processing generates cytosolic RNA:DNA hybrids under RS conditions remains to be established.

Here, the discussion focused on cytosolic DNA of nuclear origin, in connection with the RS response. However it is worth mentioning that mitochondria can also represent a significant source of inflammatory RNA and DNA fragments under conditions of DNA damage. Upon breakage of mitochondrial DNA, the RIG-I—MAVS pathway senses RNA to activate IFNs (Tigano et al., 2021). Even after γ-irradiation, part of the inflammatory response depends on mitochondrial DNA damage (Tigano et al., 2021). MRE11 deficiency can also lead to the release of mitochondrial DNA, activating the inflammasome via AIM2 and NLRP3, and mediating cell death (Li et al., 2019).

## Perspectives

Events threatening genome stability during DNA replication can produce a large amount of cytosolic DNA fragments, sensed by the cGAS-STING pathway to induce type I IFNs. This link between RS and inflammation has major implications for cancer treatment and immunotherapy. Potential targets to modulate this interplay include factors regulating the homeostasis of cytosolic DNA. For instance, TREX1 is a druggable enzyme that could be inhibited to prevent the degradation of RS-induced cytosolic DNA and promote inflammation during cancer treatment. However, the regulation of TREX1 levels and activity remain poorly understood at the molecular level. In particular, TREX1 could have nuclear or mitotic functions that need to be further investigated to ensure that its inhibition would not further increase genomic instability in cancer patients.

Cytosolic DNA results, at least in part, from the action of endo- and exonucleases. A better characterization of the substrates of MRE11, EXO1 and MUS81 at stalled forks is therefore important to understand how these enzymes contribute to inflammation. It would also be important to develop reliable methods to extract, concentrate and sequence cytosolic DNA in order to determine its origin. Indeed, under mild RS conditions, structure-specific nucleases such as MUS81 cleave replication intermediates accumulating within late-replicating regions of the genome, which should be overrepresented in cytosolic DNA. In contrast, the large chromosome fragments present in micronuclei should not display such a replication timing bias. In both cases, the ability of cells to modulate the rates of initiation and elongation in response to RS will determine the persistence of unreplicated DNA at the end of S phase and will therefore impact both the stability of the genome and the production of pro-inflammatory cytokines.

Recent evidence indicates that RS can also be caused by an increased fork velocity (Maya-Mendoza et al., 2018). Since ISG15, one of the interferon-stimulated genes induced by the cGAS-STING pathway, can also promote RS by accelerating forks in a RECQ1-dependent manner (Raso et al., 2020), it is tempting to speculate that inflammation induces RS in the same way that RS induces inflammation. This view is supported by a recent report showing a STING-dependent acceleration of replication forks in *Hidradenitis suppurativa*, a chronic inflammatory skin disease affecting hair follicle stem cells (Orvain et al., 2020). Another example of the unexpected links between RS and inflammation concerns the poor survival of female embryos that are deficient for the replicative helicase component MCM (MCM4^*chaos*3^) can be suppressed by the administration of the anti-inflammatory drug ibuprofen to gestating mice (McNairn et al., 2019). Male embryos are protected from lethality presumably through the anti-inflammatory effect of testosterone. This result suggests that RS generated *in vivo* by deregulated origin usage results in a chronic inflammatory response that compromises embryonic viability. Together, these findings indicate that the interplay between RS and inflammation has broad physiological consequences beyond cancer and aging.

## Author Contributions

HT wrote the original draft and assembled the figures. HT and PP wrote, reviewed, and edited the text and the figures. Both authors approved the submitted version of the article.

## Conflict of Interest

The authors declare that the research was conducted in the absence of any commercial or financial relationships that could be construed as a potential conflict of interest.
